# Changes in Proliferation Kinetics of T Cells: A New Predictive Cellular Biomarkers for Early Rheumatoid Arthritis?

**DOI:** 10.1007/s10875-012-9692-1

**Published:** 2012-04-25

**Authors:** Justyna Pawłowska, Żaneta Smoleńska, Zbigniew Zdrojewski, Jacek M. Witkowski, Ewa Bryl

**Affiliations:** 1 Department of Pathophysiology, Medical University of Gdańsk, Dębinki 7, 88-210 Gdańsk, Poland; 2 Department of Internal Medicine, Connective Tissue Diseases and Geriatrics, Medical University of Gdańsk, Gdańsk, Poland

**Keywords:** Early rheumatoid arthritis, undifferentiated arthritis, T cells, lymphocyte proliferation, predictive biomarkers

## Abstract

**Objective:**

It has been demonstrated that early treatment of rheumatoid arthritis (RA) patients prevents further joint damage and disability, but biomarkers enabling early RA to be distinguished within the undifferentiated arthritis (UA) cohort are still being sought.

**Purpose:**

The aim of the research was to study the pathomechanism of initiation and progression of UA→RA and to find such new predictive biomarkers on the basis of functional studies of the immune system.

**Methods:**

55 patients with UA were enrolled into the study and followed up for 2 years. The dynamic parameters of proliferation of the peripheral blood CD4+ T cells were recorded at the UA stage. During the follow-up study, standard diagnostic procedures were performed to make the final diagnosis. Comparison of the CD4+ T cell proliferation parameters in the UA-RA and UA-non-RA patients was conducted after the final diagnosis was established.

**Results:**

Our studies showed that the G0-G1 transition time, the cell cycle duration, the number of cell divisions per dividing CD4+ cells and the percentage of dividing CD4+ T cells differed significantly between UA-RA and UA-non-RA patients. Moreover, these proliferation parameters achieved higher specificity and sensitivity in the detection of early RA within UA patients compared to the routine serological tests available.

**Conclusion:**

The proliferation parameters of CD4+ T cells reflect central pathophysiological changes in RA and can be used as new biomarkers for early RA diagnosis, which would enable the international rheumatology recommendation to be achieved concerning the early diagnosis and treatment of RA patients.

## Introduction

The term “undifferentiated arthritis” (UA) is applied to the most common type of arthritis at the early stage when, in the absence of current recommended diagnostic criteria, it cannot be classified into the well-known clinical disease categories of defined inflammatory rheumatic diseases [1]. At the stage identified as UA, identification of the subset of patients destined to develop rheumatoid arthritis (RA) - the most severe and persistent form of rheumatic disease - is a challenge for both clinicians and researchers. The new diagnostic approach would allow disease-modifying anti-rheumatic drugs (DMARDs) to be introduced as an early treatment strategy [2]. The body of evidence has highlighted the effectiveness of DMARDs in patients with early RA before the first radiographic evidence of erosions, in preventing further joint damage and disability [3]. In line with European League Against Rheumatism/American College of Rheumatology recommendation, the concept of a “window of opportunity” for the treatment of the patients should be acted upon as early as possible [4]. Many studies have shown that such a therapeutic window of opportunity may exist within only the first few months of the disease [3]. The possible advantage of early therapy underscores the need for a new diagnostic tool for early diagnosis of such patients. On the other hand, clear differentiation between early RA and other rheumatic diseases at such an early stage (UA) still causes major difficulties for rheumatologists. In fact, current diagnostic criteria have not moved beyond describing the early symptoms of these diseases as the UA which is clearly insufficient.

Emerging data show that not only is RA a local joint disease, but it also involves impairment of the systemic immune system, both central, (including bone marrow [5]), and peripheral [6–9]. A relatively new concept describes premature senescence of peripheral CD4+ T cells in established RA patients, demonstrated by, for example, reduced overall proliferative capacity, shorter telomere length, decreased T-cell receptor diversity [9] and decreased Klotho expression [7].

Benefiting from the technique of precise, numerical assessment of multiple parameters of lymphocyte proliferative dynamics developed in our laboratory and already shown to detect differences between proliferation of T cells of healthy young and elderly people [6], we decided to apply it as a potential tool for early diagnostics of RA. Thus, the aim of our study was to find out if specific features of lymphocyte proliferation dynamics could be ascribed to RA and if they could offer a good diagnostic approach for distinguishing patients with early RA from those with other rheumatic diseases, early in the course of the disease, as desired for improvement of the early diagnosis according to the European Standing Committee for International Clinical Studies Including Therapeutics [4].

## Materials Methods

### Patient Population

Fifty five adult patients (50 women, 5 men) with peripheral joints manifestation were enrolled in the study. Median duration of their symptoms was 5 months. Patients included into the study did not fulfill any of the existing classification criteria for any specific rheumatic disease and were classified as UA based on the literature data. Patients with a definitive diagnosis at baseline and with a documented duration of symptoms more than 1 year were excluded from the study, as well as patients with psoriatic skin manifestation at the beginning of the disease and patients with other chronic inflammatory conditions or malignancies in the medical history.

The study was approved by the local ethics committees of Medical University of Gdańsk. All patients gave their written informed consent.

### Clinical Assessment

The clinical assessment was done first at the stage of UA. Disease activity was measured by DAS28 based on the number of swollen and tender peripheral joints, patients` overall assessment by visual analogue scale (VAS) and erythrocyte sedimentation rate (ESR) before any treatment with DMARDs and/or glucocorticoids were introduced. Additionally, 24 h before the clinical assessment patients had not received any non-steroidal anti-inflammatory drugs or paracetamol.

**Fig. 1:**
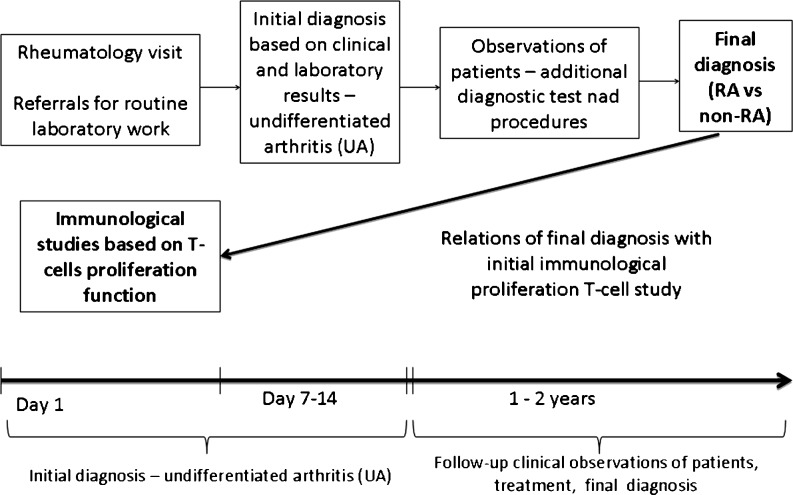
Perspective 2-year follow-up study design. Patients with UA were enrolled into the study and followed up for to 2 years. The T-cell proliferation parameters were recorded at the UA stage and before any treatment with disease-modifying anti-rheumatic drugs (DMARDs)/or glucocorticoids were introduced. Twenty four hours before the diagnostic procedure patients had not received any non-steroidal anti-inflammatory drugs or paracetamol. During the follow-up study, standard diagnostic procedures were performed to make the final diagnosis. Comparison of the results from the immunological studies was conducted after the final diagnosis – RA, non-RA was established (UA-RA vs. UA-non-RA)

Patients were followed-up by the same rheumatologist from 1 to maximum 2 years, the final diagnosis was established during that time. The design of study is shown on Fig. 1.

The final diagnosis was established according to the following criteria, available during the study: RA - American College of Rheumatology 1987 criteria [10], psoriatic arthritis - the classification criteria for Psoriatic Arthritis [11]; group of spondyloarthropathies with peripheral joints involvement including patients belong to either spondyloarthritis positive for HLA-B27 antigen or reactive arthritis based on the European Spondylarthropathy Study Group criteria [12]; primary Sjögren syndrome according to American-European Consensus Group Classification criteria [13], osteoarthritis - according to American College of Rheumatology criteria [14]. High levels of antibodies against thyreoglobulin and thyroid peroxidase after exclusion of Sjögren syndrome were classified as polyarthralgia-associated thyroiditis. When the definitive diagnosis after the follow-up study was not established, patients were classified as early UA. Patients who initially presented features of RA, but who subsequently developed clinical and serological characteristics of systemic erythematosus lupus (SLE) were diagnosed as a rhupus syndrome, defined as an “overlap syndrome” of systemic erythematosus lupus (SLE) and RA with positive for RF and/or anti-CCP and anti-double-stranded DNA antibodies (anti-dsDNA) [15]. Final diagnosis, especially of RA was supported by X-ray and ultrasound joint examination.

The patients who developed RA were eventually grouped in UA-RA subgroup, while patients who developed other form of rheumatic diseases were grouped as UA-non-RA subgroup.

### Laboratory Diagnostic Procedures

The laboratory diagnostic procedures were performed according to the recommendation by standard routine procedures including: ESR, anti-CCP, RF, anti-nuclear (ANA-Hep2 antibodies), SSA/Ro, SSB/La, anti-double-stranded DNA antibodies (ds-DNA) and HLA-B27 antigen. RF was determined by immunoturbidimetric assay (Abbott), anti-CCP antibodies were determined by enzyme-linked immunoadsorbent assay ELISA (Imtec Immunodiagnostica GmbH). ANA-Hep2 antibodies were measured by indirect fluorescent antibody (Medipan GmbH) while HLA-B27 by PCR electrophoresis kit (Medipan GmbH). Quantitative analysis of antibodies: anti-SSA, anti-SSB, anti-dsDNA antibodies was done by commercially available ELISA kits (Euroimmun).

### Ex Vivo Lymphocyte Study

The proportion and absolute number of peripheral leucocytes and lymphocytes, as well as proportion and absolute number of CD3^+^CD4^+^ ex vivo before proliferation analysis were measured by flow cytometry, using fluorescently tagged anti-CD3 and anti-CD4 monoclonal antibodies (DAKO, Glostrup, Denmark).

### Assessment of Peripheral Blood Lymphocyte Proliferation Dynamics

Venous blood samples were collected at the first visit to rheumatologist, before standard diagnostic procedure and before any treatment with DMARDs and/or glucocorticoids were introduced. Twenty four hours before the diagnostic procedure patients had not received any non-steroidal anti-inflammatory drugs or paracetamol.

The test was performed according to literature [16]. Briefly, peripheral blood mononuclear cells (PBMC) were isolated by Histopaque™ flotation. PBMC were stained with 10 μM carboxyfluorescein diacetate succinimidyl ester (CFSE, Molecular Probes-Eugene, Oregon). CFSE-loaded cells were suspended in RPMI 1640 supplemented with 10 % fetal bovine serum, 2 mM -glutamine, penicillin and streptomycin (Sigma, St Louis, MO) and samples of 1 × 10^6^ PBMC × ml^−1^ were stimulated with 0.2 μg immobilized anti-CD3 antibody (OKT3, BD Biosciences Pharmingen, San Diego, CA) in culture plates for up to 118 h, in 5 % CO_2_ at 37°C. Cultured PBMC were collected at two time points (72 and 118 h) and stained with monoclonal antibodies against surface T-cells markers. Single parameter histograms of CFSE staining intensity were generated for T-cells subpopulation. Numbers of cells in each peak were determined with the help of WinMDI™ v. 2.09 (J. Trotter, the Scripps Research Institute, La Jolla, CA). The cell cycle duration described as the length of a single cell division [hour], the length of G0-G1 transition time [hour] defined as the period from the onset of stimulation to the beginning of the gap 1 (G1 phase) of the interphase (duration of pre-division resting phase), the number of cell divisions per dividing cells and the percentage of dividing cells calculated as ratio of the values obtained after 72 and after 112 h of the cell cultivation were calculated according to mathematical formula [16]. The number of divisions calculated per dividing cells is defined as the sum of divisions required to produce the observed numbers of cells in all generations divided by the sum of dividing cells while the percentage of dividing cells is defined as cells that divided in response to the stimulation, producing viable progeny at the end of observation period.

### Statistical Analysis

The differences between analyzed clinical and laboratory parameters were evaluated between UA patients who developed RA (UA-RA) and patients who developed other rheumatic diseases (UA-non-RA) subgroups. The significances of differences between quantitative variables were calculated by U-Mann–Whitney test, while of those observed between qualitative variables by test of difference between two structure factors. Correlations between two quantitative variables were performed by Spearman`s rang correlation test and presented by Spearman`s correlation coefficients ρ.

All tabularized results are presented as medians with 25th and 75th quartile, while graphical illustration (box-and-whisker plots) included additionally the minima and maxima observed. Univariate logistic regression analysis was performed to identify relevant variables for distinguishing UA-RA from UA-non-RA subgroup. Then, multiple regression analysis was performed including only the significant variables from the univariate logistic analysis. *p* < 0.05 was considered significant, while 0.10 ≥ p > 0.05 was considered borderline statistical significant. Diagnostic test properties (sensitivity and specificity) were calculated for each proliferation parameter and serological tests based on the final diagnosis of UA cohort and additionally for the proliferation parameters on the cut-off value evaluated based on receiver operating characteristic curves (ROC). ROC was created for the proliferation parameters while the cell cycle duration time and the ratio of number of cell divisions and the ratio of percentage of dividing cells were classified as a destimulant value. Area under the curve (AUC) was additionally calculated. Statistical analysis was performed using Statistica 9 StatSoft Poland.

## Results

### Basic Clinical Characteristics at Baseline Divided According to Final Diagnosis and Comparison Between UA-RA and UA-non RA

During clinical 2-year prospective follow-up study of 55 UA patients (Fig. 1), 50 (91 %) of them fulfilled the diagnostic criteria for specific rheumatic disease including: RA, primary Sjögren`s syndrome, psoriatic arthritis, polyarthralgia-associated thyroiditis, spondyloarthritis positive for human histocompatibility leukocyte B27 antigen (HLA-B27) antigen, reactive arthritis, rhupus and osteoarthritis. Remaining 5 (9 %) of the patients did not fulfilled any specific criteria even after 2 years of observation and was categorized as early UA. 10 female patients (18 %) developed RA during the time of observation. Basic clinical characteristics at baseline were compared between early RA (UA-RA) and patients who developed other rheumatic diseases (UA-non-RA), divided according to final diagnosis (Table I). The subgroups did not differed in age or percentage of female patients. UA-RA patients achieved only borderline statistically significant higher disease activity score (DAS28) value than UA-non-RA subgroup during the first visit (*p* = 0.09).

**Table I:** Basic clinical, laboratory and immunological differences between UA patients subgroups divided according to final diagnosis

	UA-RA (*n* = 10)	UA-non-RA (*n* = 45)	p ^a^
Basic characteristic			
* Age*, years, median (IQR)	54 (46–57)	46 (40–54)	0.169
* Gender*, number of females/men	10/0	40/5	0.136
Activity components			
* Number of tender joints*, median (IQR)	7 (5–11)	6 (3–10)	0.380
* Number of swollen joints*, median (IQR)	4 (1–6)	2 (1–5)	0.360
* VAS score by patients*, median (IQR)	6.5 (6–7)	5.0 (4–6)	0.067
* ESR*, [mm/hour], median (IQR)	25 (22–48)	22 (11–35)	0.335
* DAS28*, median (IQR)	5.31 (4.30–5.97)	4.54 (3.38–5.33)	0.093
Immunological characteristic			
* Absolute number of leucocytes* [g/L],median (IQR)	6.55 (4.27–8.31)	6.37 (4.82–7.84)	0.679
* % of lymphocyte,* median (IQR)	30.00 (27.40–37.13)	29.75 (21.25–35.85)	0.464
* Absolute number of lymphocyte* [g/L], median (IQR)	1.84 (1.55–2.12)	1.79 (1.31–2.12)	0.631
* % of CD3 + CD4+,* median (IQR)	58.41 (46.90–63.87)	60.81 (55.91–65.81)	0.270
* Absolute number of CD3 + CD4+* [g/L], median (IQR)	0.97 (0.78–1.32)	0.96 (0.79–1.38)	0.887
* RF*, positive, %	80 %	43 %	0.078
* anti-CCP*, positive, %	80 %	33 %	0.025
* ANA-Hep2*, positive, %	0 %	49 %	-
Proliferation dynamics parameters			
* Cell cycle duration* [h]	20.36 (19.45–24.89)	30.73 (27.03–37.31)	<0.001
* G0-G1 transition time* [h]	32.76 (27.64–42.39)	0.5 (0–17.89)	<0.001
* Ratio of number of cell divisions*	0.67 (0.61–0.84)	0.87 (0.82–0.94)	<0.001
* Ratio of percentage of dividing cells*	0.73 (0.68–0.82)	0.95 (0.88–0.98)	<0.001

^a^ The differences between quantitative variables were calculated by U-Mann–Whitney test, while between qualitative variables by test of difference between two structure factors

VAS- visual analogue scale, ESR-erythrocyte sedimentation rate, DAS – disease activity score, RF - rheumatoid arthritis, anti-CCP - anti-cyclic citrullinated proteins ANA-antinuclear antibodies

### Immunological Characteristics of UA Patients Divided According to Final Diagnosis and Comparison Between UA-RA and UA-non RA

We compared immunological characteristics recorded in UA patients between UA-RA and UA-non-RA subgroups divided according to final diagnosis. We did not disclose statistical significance differences in number of lymphocytes between UA-RA and UA-non-RA patients. We showed differences between percentages of positive anti-cyclic citrullinated proteins antibodies (anti-CCP) patients between the subgroups. Basic immunological characteristic for enrolled patients are shown in Table I.

### Differences in CD4+ Lymphocyte Proliferation Dynamics Between UA-RA and UA-non-RA Subgroups

**Fig. 2:**
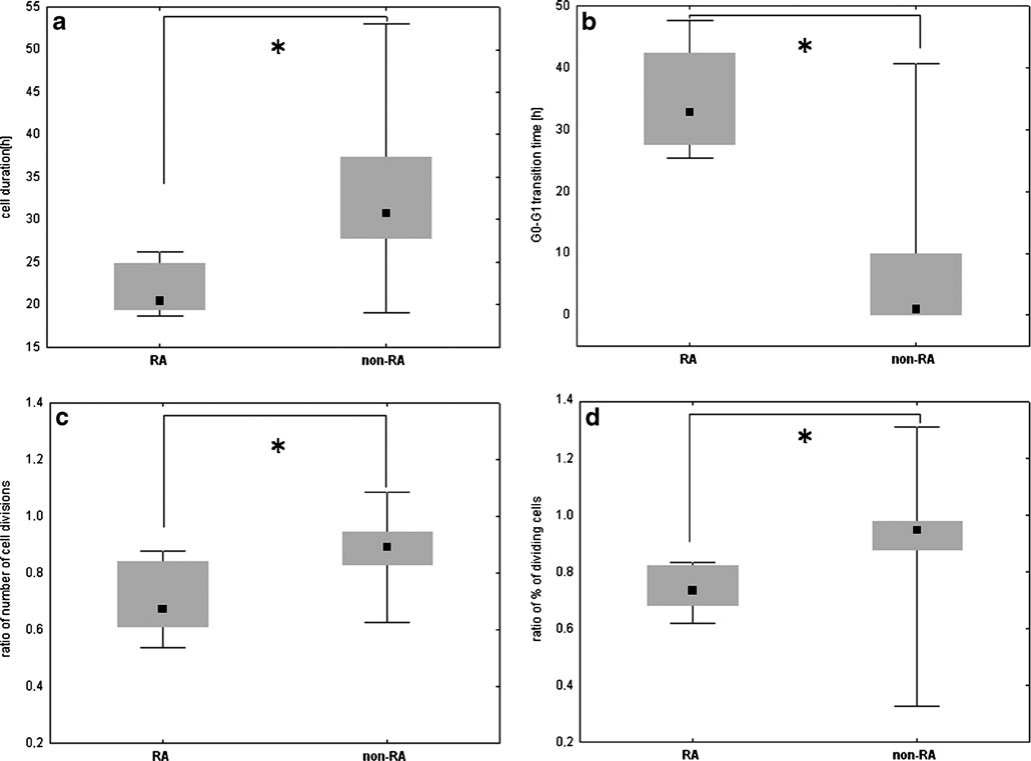
Comparison of the proliferation dynamics parameters between UA-RA and UA-non-RA patients. Each of the calculated proliferation parameters differed significantly between the subgroups divided according to the final diagnosis – RA and non-RA. (**a**) Cell cycle duration. (**b**) G0-G1 transition time. (**c**) Ratio of number of cell divisions. (**d**) Ratio of percentage of dividing cells. The cell cycle duration described as the length of a single cell division [hour], the length of G0-G1 transition time [hour] defined as the period from the onset of stimulation to the beginning of the gap 1 (G1 phase) of the interphase, the ratio of number of cell divisions defined as the sum of divisions required to produce the observed numbers of cells in all generations divided by the sum of dividing cells and the ratio of percentage of dividing cells defined as cells that divided in response to the stimulation, and were calculated according to the mathematical formula. The results are presented in box-and-whisker plots using medians and 25th and 75th quartile with whiskers to the minima and maxima of the data. * - statistical significance (*p* < 0.001) using U-Mann–Whitney test

Differences in CD4+ lymphocyte proliferation dynamics between UA-RA and UA-non-RA subgroups were indicated (Table I). The proliferation parameters differed significantly (*p* < 0,001) between UA-non RA and UA-RA patients regarding the cell cycle duration (Fig. 2a), the G0-G1 transition time (Fig. 2b), the ratio of number of cells divisions per dividing cells (Fig. 2c) and the ratio of percentage of dividing cells (Fig. 2d). The ratio of number of cell divisions per dividing cells and the ratio of percentage of dividing cells were characterized by the higher diagnostic test properties than each of the parameters alone (not shown). The highest sensitivity and specificity of early RA detection were indicated for the proliferation biomarkers, compared to the laboratory serological tests performed at standard diagnostics (Table II).

**Table II:** Diagnostic test efficacy of RF, anti-CCP and the proliferation parameters in prediction of early RA

Laboratory parameter	AUC (95 % CI)	specificity, % (95 % CI)	sensitivity, % (95 % CI)
Standard serological test			
RF	-	56.3 (39.3–71.8) ^a^	70.0 (39.7–89.2) ^a^
Anti-CCP	-	66.7 (52.1–78.6) ^a^	80.0 (49.0–94.3) ^a^
ANA-Hep2	-	51.1 (37.1–64.9) ^a^	4.5 (5.0–32.1) ^a^
Proliferation parameters			
Cell cycle duration	0.948 (0.888–1.000)	92.5 (80.1–97.4) ^b^	80.0 (49.0–94.3) ^b^
G0-G1 transition time	0.944 (0.876–1.000)	89.0 (75.9–95.4) ^b^	95.5 (67.9–99.5) ^b^
Ratio of number of cell divisions	0.848 (0.713–0.982)	97.5 (87.1–99.6) ^b^	60.0 (31.3–83.2) ^b^
Ratio of percentage of dividing cells	0.818 (0.703–0.932)	74.1 (58.9–85.1) ^b^	95.5 (67.9–99.5) ^b^

Data is presented as 95% confidence interval

^a^ The classification parameters were calculated after final diagnosis was established

^b^ The classification parameters were calculated after final diagnosis was established based on the cut-off value evaluated based on ROC plot

AUC: area under the curve, RF: rheumatoid arthritis, anti-CCP: anti-cyclic citrullinated proteins, ANA: antinuclear antibodies

### Identifying Relevant Variables Distinguishing UA-RA from UA-non-RA Subgroups

**Fig. 3:**
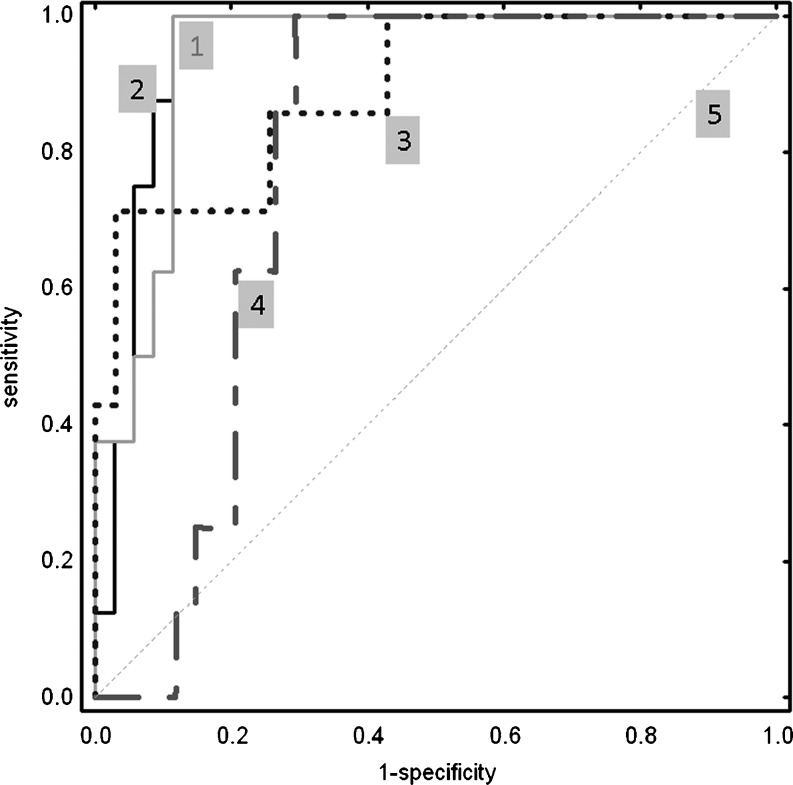
Receiver operating characteristics curves of the proliferation parameters. In order to compare diagnostic efficacy of the proliferation parameters for distinguish early RA among UA patients, ROC plot was created. The proliferation parameters achieved high AUC values wherein the G0-G1 transition time and the cell cycle duration were the most significantly strong predictive biomarkers for prediction of UA→RA progression. (1) G0-G1 transition time. (2) Cell cycle duration time. (3) Ratio of number of cell divisions per dividing cells. (4) Ratio of %percentage of dividing cells. (5) Regarding line. The cell cycle duration, the ratio of number of cell divisions and the ratio of percentage of dividing cells were presented as a destimulant variables

Among all considered factors included: age, gender, component of disease activity, diagnostic results, the proliferation parameters, we identified relevant variables, which were included in the multiple regression analysis. Neither age, gender nor the disease activity factors possessed significant predictive power. Only the proliferation parameters, anti-CCP and rheumatoid factor (RF) positivity allowed distinguishing between UA patients destined to develop of RA from the non-RA subgroup with high statistical significance. Among the relevant variables, only the G0-G1 transition time and the cell cycle duration were significantly strong parameters for early RA distinguish (*p* < 0.05) as well the ratio of number of cell divisions per dividing cells and the ratio of percentage of dividing cells (*p* = 0.09). The highest values of AUC were recorded for the G0-G1 transition time and the cell cycle duration (Fig. 3).

### Correlation Between the Proliferation Dynamics Parameters and Disease Activity in UA-RA Subgroup

In order to find out if the proliferation parameters associate with severity of RA we correlated each of the parameter with DAS28 values assessed at the stage of UA. In subgroup of UA-RA we indicated that there was a statistical significant positive correlation between the G0-G1 transition time and DAS28 values (Spearman`s correlation coefficient ρ = 0.44, *p* < 0.05). The opposite correlation was showed for the cell cycle duration and DAS28 values, but this correlation did not reach statistical significance (Spearman`s correlation coefficient ρ = − 0.23, *p* > 0.05). Neither the ratio of number of cells divisions per dividing cells nor the ratio of percentage of dividing cells correlate with DAS28 values (accordingly Spearman`s correlation coefficients ρ = 0.08 and ρ = 0.02, respectively).

## Discussion

Firm diagnosis of RA obtained prior to the onset of severe disability and irreversible morphological damage to cartilage and bone is of the utmost importance [17]. From onwards 1987 the limitations of the American College of Rheumatology criteria for early RA diagnosis resulted in the establishment of new classification criteria by European League Against Rheumatism/American College of Rheumatology experts in 2010 with the aim of identifying patients with inflammatory arthritis who are at high risk of developing persistent and erosive RA [18]. These criteria are still classification rather than diagnostic criteria, and therefore diagnostic methods which could be termed as biomarkers for such an early distinction are still missing.

Among the available laboratory markers for RA are the recommendations of the 2010 European League Against Rheumatism/American College of Rheumatology which include, in addition to RF, anti-citrullinated peptide antibodies (ACPA), mainly represented as anti-CCP antibodies. Many results have been obtained which confirm that anti-CCP antibodies have higher specificity and sensitivity than RF in the diagnosis of RA. On the other hand, a number of reports have shown great disproportion in the sensitivity and specificity calculations of serological markers in terms of providing an accurate prognosis of early RA. In addition, the results in diagnosis and prediction of early RA have been sharply contradictory and differed from those for established RA [19–21]. According to the published results the sensitivity of anti-CCP antibodies in early RA ranges from 39 to 61 %, while the specificity ranges from 93 to 98 % [20, 21]. Similarly the RF marker showed a sensitivity of 31 to 54 % with a specificity from 86 to 93 % [20–22]. The great disparity between the results for sensitivity and specificity is mainly due to the discrepancies in methodology for the kits that are commercially available and differences in set cut-off values and test generation. Despite the higher specificity of the anti-CCP test compared to RF in the diagnosis of early RA, RF still seems to be useful in diagnosis. Ursum et al. showed that RF may be present in patients with RA, the serological negative of anti-CCP antibodies [23]. However, RF and anti-CCP antibodies are detected even in subjects with non-rheumatic disease [23, 24]. One of the severe limitations of anti-CCP antibodies was demonstrated by Ioan-Facsinay et al. [25], who showed that anti-CCP antibodies as measured by the most frequently performed test using the ELISA second generation set of the test and specific anti-CCP antibodies are largely cross-reactive with many epitopes, which outweighs their heterogeneity. These results hardly make either marker acceptable as an early RA predictor.

Our study revealed that 80 % of early RA patients were positive for anti-CCP antibodies and 70 % for RF, but that antibodies were very frequently presented also in other subgroups of very early rheumatic diseases, which decreased their predictive value. The specificities of the RF and anti-CCP were therefore much lower for our patients than those cited above. It is important to stress that there are many limitations of calculations concerning the diagnostic accuracy of the markers, including differences between studies in terms of the population analyzed, the frequent lack of clear definition of the participation of those with disease (e.g. diseases duration), differences in the methods of validating the diagnostic accuracy or lack of internal standardization [26]. In line with this, many of the calculations of the diagnostic accuracy of the anti-CCP antibodies and RF markers are available are calculated on the basis of patients with established and early rheumatic disease and with inclusion of healthy subjects. The specificity of the markers in such studies may therefore be underestimated in comparison with that calculated here and in the studies which included only very early arthritis group [26, 27]. In our study the specificity and sensitivity calculations for anti-CCP antibodies, RF, ANA-Hep2 and proliferation parameters are based only on very early UA-subgroups that were characterized by very similar clinical presentation, while the healthy cohort was not included.

In addition, it should be pointed out that citrullination was observed also during unrelated inflammation and apoptosis, which emphasized that the presence of citrullinated proteins at the early stage of immune diseases may not be specific for RA only [28]. This is in keeping with the marked presence of anti-CCP antibodies demonstrated by us among UA patients at the earliest stage of disease. The other possible explanation of the low specificity of anti-CCP antibodies and RF in our patient cohort in comparison with other published results might be the relatively large number of patients with primary Sjögren syndrome, psoriatic arthritis and thyroid autoimmune diseases developed from our initial group of patients with UA who manifested arthritis at the beginning of the follow-up study. The presence of RF as well as anti-CCP antibodies in other rheumatic patients, especially those with psoriatic arthritis and systemic erythematosus lupus (SLE), has been confirmed by other researchers [29, 30]. On the other hand, our study did not include patients with other forms of rheumatic diseases such as Wegeners`s granulomatosis or viral- induced arthritis included in other cited studies.

The low diagnostic power of the serological markers available and recommended was confirmed in our study in the regression analysis, which showed that the proliferation parameters are stronger factors for distinguishing the UA-RA group from the UA-non-RA group. Despite the observation that some disease activity components differed in UA patients developing other forms of rheumatic disease, neither the disease activity (DAS28) parameter nor its components were useful for distinction of the UA-RA from the UA-non-RA subgroup.

Therefore, we undertook the challenge of meeting the need for more sensitive and specific predictors of UA → RA transition. Our idea came from the hypothesis concerning the premature immunosenescence of CD4+ T cells in RA patients. Having established a platform to study proliferative dynamics of these cells including previously unavailable parameters before [6], we decided to check if any of these could serve as new biomarkers for early RA with possibly higher predictive power than anti-CCP and RF.

Our results confirmed that the proliferation status of CD4+ T cells in early RA patients reflects their (suggested) premature immunosenescence. We have shown before that the cell cycle of CD4^+^ cells of healthy elderly donors stimulated with anti-CD3 and anti- CD28 antibodies is significantly shorter, while the G0-G1 transition time is extended compared to that found in young people [6]. Thus, different characteristics of the dynamic parameters of the cell cycle in early UA-RA patients and in non-UA-RA patients shown in our study confirm the immunosenescence hypothesis of RA [9, 31]. In addition, our preliminary observation showed that patients from UA group who developed RA, and did not achieved remission after DMARDs treatment for 6 months – 1 year, still had prolonged G0-G1 transition time and shorter the length of the cell cycle duration (own observation).

Important issue which remains to be discussed is in particular whether the T cells alterations we have exposed vary between the genders. Compelling evidence obtained by many authors demonstrates that there is a significant female excess for the major connective tissue autoimmune disease [32]. In our study only female patients developed RA which strongly confirms this trend. Nevertheless, in the group of UA-non-RA we did not show any differences in all proliferation parameters between male and female patients, what might suggest that the biomarkers proposed by us did not change depending the genders.

The caveat that must be attached to our study is that a relatively small group of patients was enrolled in the investigation. However, the intensive laboratory work and 2 years of follow-up from the UA stage to the final diagnosis for 55 patients is, in our opinion, a sufficient basis for suggesting the new biomarkers in particular as the parameters proposed were the most significant of various factors, including serological markers and disease activity features, in distinguishing RA from early UA at the outset of the diagnostic procedure very early in the course of the disease. The method underwent the process of validation and laboratory verification.

## Conclusions

We have thus proposed, for the first time, a set of new biomarkers related to the proliferation kinetics of CD4+ T cells for highly specific and sensitive prediction as to which early UA patients will progress to RA. In addition to the high degree of sensitivity and specificity of the new biomarkers, the proliferation parameters reflect central pathophysiological changes in early RA, emphasizing the value of the new biomarkers in comparison with RF and anti-CCP antibodies.
